# Amitriptyline Decreases GABAergic Transmission in Basal Forebrain Neurons Using an Optogenetic Model of Aging

**DOI:** 10.3389/fnagi.2021.673155

**Published:** 2021-05-28

**Authors:** Eunyoung Bang, Angelika Tobery, Karienn S. Montgomery, Annette S. Fincher, David J. Earnest, David A. Murchison, William H. Griffith

**Affiliations:** Department of Neuroscience and Experimental Therapeutics, College of Medicine, Texas A&M University Health Science Center, Bryan, TX, United States

**Keywords:** amitriptyline, synaptic, patch-clamp, quantal analysis, optogenetics, basal forebrain, circadian, aging

## Abstract

The antidepressant drug amitriptyline is used in the treatment of clinical depression and a variety of neurological conditions such as anxiety, neuropathic pain disorders and migraine. Antidepressants are associated with both therapeutic and untoward effects, and their use in the elderly has tripled since the mid-1990s. Because of this widespread use, we are interested in testing the acute effects of amitriptyline on synaptic transmission at therapeutic concentrations well below those that block voltage-gated calcium channels. We found that 3 μM amitriptyline reduced the frequency of spontaneous GABAergic inhibitory postsynaptic currents (IPSCs) and reduced quantal content in mice at ages of 7–10 mo. and 23–25 mo., suggesting a presynaptic mechanism of action that does not diminish with age. We employed a reduced synaptic preparation of the basal forebrain (BF) and a new optogenetic aging model utilizing a bacterial artificial chromosome (BAC) transgenic mouse line with stable expression of the channelrhodopsin-2 (ChR2) variant H134R specific for GABAergic neurons [VGAT-ChR2(H134R)-EYFP]. This model enables optogenetic light stimulation of specific GABAergic synaptic terminals across aging. Age-related impairment of circadian behavior was used to confirm predictable age-related changes associated with this model. Our results suggest that low concentrations of amitriptyline act presynaptically to reduce neurotransmitter release and that this action is maintained during aging.

## Introduction

Amitriptyline (AMI) is a tricyclic antidepressant used for various neurological disorders such as anxiety, depression, neuropathic pain disorders and migraine (Baldessarini, 2006; Couch et al., 2011; Moore et al., 2015; Urquhart et al., 2018). Because of its diverse therapeutic applications, AMI continues to be used throughout life span, and overall antidepressant use among patients over 65 years of age has more than tripled from 1995 to 2005 (National Center for Health Statistics, 2007). Although the actions of AMI to block reuptake of norepinephrine and serotonin have been studied for many years, much less is known about the additional cellular mechanisms of AMI that may contribute to its therapeutic efficacy. For example, AMI acts to inhibit ion channels such as the voltage-gated sodium, calcium and potassium channels (Nicholson et al., 2002; Yan et al., 2010; Wu et al., 2012). AMI also acts as an antagonist of the serotonin, the α1-adrenergic, the histamine, and the muscarinic acetylcholine receptors (Richelson, 1979; Kachur et al., 1988; Nojimoto et al., 2010; Pandey et al., 2010; Liu et al., 2012). Moreover, it possesses neurotrophic activity by acting as an agonist of TrkA and TrkB receptor which BDNF activates (Jang et al., 2009). Many, but not all, of these actions to block ion channels occur at higher than therapeutic concentrations of approximately 3–6 μM (Glotzbach and Preskorn, 1982; Lavoie et al., 1990; Baldessarini, 2006). Importantly chronic low dose AMI, within the therapeutic range, is an effective therapy for lower back pain (Urquhart et al., 2018), reducing nicotine-induced increase in C-fiber excitability in humans (Freysoldt et al., 2009), and prevention of age-related impairment in the water maze task in rats (Yau et al., 2002). However, some human studies demonstrate the negative effects of AMI on cognitive function within the therapeutic ranges of depression treatment (Peck et al., 1979; Branconnier et al., 1982; Linnoila et al., 1983; Curran et al., 1988). Therefore, the purpose of the present study is to investigate whether therapeutic concentrations of AMI interfere with synaptic transmission in the basal forebrain (BF) using a new synaptic optogenetic model and whether these actions are maintained during aging.

The BF contains a heterogeneous population of cholinergic and non-cholinergic neurons located in the medial septum, diagonal band, nucleus basalis, ventral pallidum, substantia innominata, globus pallidus and the internal capsule (Wainer et al., 1985; Zaborszky et al., 1999; Hajszan et al., 2004; Manseau et al., 2005). These neurons play an important role in cognitive functions such as attention, arousal, and memory (see review, Záborszky et al., 2018) and our lab has been studying calcium homeostasis, voltage-gated calcium currents and synaptic transmission in these neurons across aging for many years (Griffith and Murchison, 1995; Murchison and Griffith, 1996, 1998, 2007; Murchison et al., 2009). Using a reduced synaptic preparation, we have shown that decreased GABAergic inhibitory synaptic transmission in BF is associated with age-related cognitive impairment in rats (Griffith et al., 2014). We have recently reproduced many of these earlier results in a new optogenetic aging model utilizing a bacterial artificial chromosome (BAC) transgenic mouse line with stable expression of the channelrhodopsin-2 (ChR2) variant H134R specific for GABAergic neurons [VGAT-ChR2(H134R)-EYFP] (Montgomery et al., in submission). In the present study, we have used this optogenetic aging model with whole-cell voltage-clamp, Ca^2+^-sensitive fluorescent imaging and the reduced synaptic preparation to examine the effect of AMI on inhibitory synaptic transmission in the BF. The great advantage of the reduced synaptic preparation is that it is possible to selectively stimulate GABAergic nerve terminals on well-clamped isolated BF neurons. This model allows us to calculate relative changes in quantal content (m) of transmitter release and identify presumed presynaptic functions. Finally, age-related changes in circadian wheel-running activity were used to further characterize the VGAT optogenetic mouse as a viable aging model.

## Materials and Methods

### Animals and Treatments

Preliminary experiments to construct a concentration response curve for AMI inhibition of high voltage-activated (HVA) calcium currents utilized juvenile Fischer 344 rats. We have experience studying the physiology and pharmacology of Ca^2+^ currents in basal forebrain neurons of Fischer 344 rats (Murchison and Griffith, 1996, 1998), and therefore, used juvenile rats to confirm the concentration range for Ca^2+^ current inhibition by AMI. Pregnant F344 dams were obtained (Harlan, Indianapolis, IN) and allowed to give birth. Juveniles of both sexes were used between 10 and 30 days of age. For all other experiments we used VGAT-ChR2(H134R)-EYFP and wildtype (WT) C57 Bl/6 mice. Breeding pairs were purchased from Jackson Labs (Stock 014548; Zhao et al., 2011) and we established an aging colony where mice of all ages and both genotypes could be obtained. Mice were identified as WT or VGAT by standard tail-clip genotyping methods after weaning. Genotyping was done on a MJ PTC-100 Thermocycler (Bio-Rad Laboratories, Hercules, CA) using the EZ BioResearch Fast Tissue/Tail PCR Genotyping Kit (EZ BioResearch, St. Louis, MO) with value oligo primers from Thermo Fisher Scientific (Waltham, MA) and sequence from Jackson Lab (Bar Harbor, ME): transgene (∼400 bp): forward primer 11678: 5′-ACC CTT CTG TCC TTT TCT CC-3′, reverse primer 10493: 5′-GCA AGG TAG AGC ATA GAG GG-3′ and internal control (324 bp): forward primer oIMR7338: 5′-CTA GGC CAC AGA ATT GAA AGA TCT-3′, reverse primer oIMR7339: 5′-GTA GGT GGA AAT TCT AGC ATC C-3′. Samples were run with Bulldog Bioflex S50 DNA ladder (Portsmouth, NH) on 2% Agarose gel using 1 X TBE buffer with SYBR Safe DNA gel stain for 1.5 h and imaged either on a Flourchem Q or an Accuris Smartdoc.

For the data in Figure 3, to determine the concentration response relationship for AMI inhibition of spontaneous synaptic transmission, young mice were between 2 and 7 months of age (*N* = 23). For all other studies for age and AMI comparisons (Figures 4–6) all mice underwent circadian wheel-running prior to drug testing. For wheel-running behavior, young wildtype (WT, *N* = 6) and VGAT (*N* = 6) mice were 7–10 months while aged WT (*N* = 10) and VGAT (*N* = 10) mice were 23–25 months. “N” refers to the number of mice, while “n” is the number of neurons. All rodents were maintained in the AAALAC-accredited vivarium at the Texas A&M University Health Science Center under controlled conditions (22–25°C; lights 0700–1900 h; mouse chow and water ad lib) in accordance with policies of the Texas A&M University Laboratory Animal Care Committee and NIH guidelines. TAMU animal protocol number 2019–0362.

### Immunohistochemistry

Mice were deeply anesthetized with isoflurane and transcardially perfused with ice-cold phosphate-buffered saline (PBS, 0.01 M, pH 7.4) followed by a fixative, 4% paraformaldehyde (PFA) in PBS. Brains were quickly removed from the skull and post-fixed overnight in the same solution at 4°C followed by dehydration in 30% sucrose solution overnight at 4°C. The brain was cut into 40-μm coronal sections using a freezing microtome. Sections were washed 3 times in Tris-buffered saline (TBS, 100 mM Tris-HCl, 150 mM NaCl, pH 7.5) and then permeabilized with TBST (0.1% Triton X-100 in TBS) for 30 min at room temperature (RT). After permeabilization, sections were blocked (1.5 h at RT) with 5% normal donkey serum in TBST and were incubated in primary antibody with blocking solution overnight at 4°C. After incubation with primary antibody, sections were washed in TBS three times and incubated in secondary antibody with blocking solution for 2 h at RT followed by three-time wash in TBS. Lastly, they were mounted in Vectashield (Vector Laboratories, Burlingame, CA) and examined under a confocal laser-scanning microscope (FV1,200, Olympus, Shinjuku City, Tokyo). Antibodies targeting the following proteins were used: GFP (1:5,000; Abcam, Cambridge, MA) and NeuN (1:1,000; Millipore, Burlington, MA). The Alexa Fluor 488-conjugated goat anti-chicken IgY (H + L) antibody (1:300; Invitrogen, Carlsbad, CA) and Alexa Fluor 647-conjugated donkey anti-mouse IgG (H + L) antibody (1:300; Invitrogen, Carlsbad, CA) were used for secondary antibodies.

### Circadian Wheel Running Measurements

For circadian measurements of wheel- running activity, mice were housed individually in cages equipped with running wheels. Animals were maintained on a standard 12 h light/12 h dark cycle (LD 12:12; lights-on at 0600 h) and their circadian rhythms of wheel-running were continuously recorded for 30 days. Revolutions were summed and stored in 10-min bins, graphically depicted in actograms and analyzed using a computer running ClockLab data collection and analysis software (ActiMetrics, Evanston, IL). Entrainment and qualitative parameters of the activity rhythm were measured over the same interval for all animals. During entrainment to LD 12:12, the onset of activity for a given cycle was identified as the first bin during which an animal attained 10% of peak running-wheel revolutions (i.e. intensity). To measure angle of entrainment, least squares analyses was used to establish a regression line through the daily onsets of activity during the period of entrainment (30 days), and then the number of minutes before (positive) or after (negative) the time of lights-off in the LD cycle (1,800 h) was determined for each animal. Total daily activity was calculated by averaging the number of wheel revolutions per 24 h over the 30-day interval of analysis.

### Acutely Dissociated Neurons and Reduced Synaptic Preparation

Rats and mice, were decapitated after isoflurane anesthetization and brain slices (400 μm for rats, 320 μm for mice) were micro dissected to isolate the medial septum and nucleus of the diagonal band (MS/nDB), as described previously (Murchison and Griffith, 1996; Griffith et al., 2014). This tissue was kept suspended in a holding solution (mM): 140 NaCl, 2.7 KCl, 0.5 CaCl_2_, 1.2 MgCl_2_, 10 HEPES, and 33 D-glucose (pH 7.4 with NaOH, 310–330 mOsm) by a small magnetic stir bar and was continuously oxygenated with 100% O_2_ in a holding chamber. Individual tissue pieces were triturated in a pair of fire-polished glass pipettes, and the dissociated cells were dispersed and allowed to settle onto the glass floor of a recording chamber containing bath solution (mM): 140 NaCl, 2.7 KCl, 2 CaCl_2_, 1.2 MgCl_2_, 10 HEPES, and 33 D-glucose, then perfused at a rate of about 2 ml/min. Experiments were performed at room temperature within 5 h of dispersal. For the rat whole-cell Ca^2+^-current experiments and mouse fura-2 experiments depicted in Figure 2, the pieces of BF slices were treated with trypsin (Sigma, type XI or type I, St. Louis, MO) as described previously (Murchison and Griffith, 1996, 1998). Importantly, for the synaptic studies in mice, no enzyme was used prior to dissociation in order to preserve presynaptic terminals on the reduced synaptic preparation (Griffith et al., 2014). This method is similar to that described by Akaike and Moorhouse (2003) to obtain a nerve-bouton preparation, however we have refined this technique to include aging neurons.

### Electrophysiology

Whole-cell and perforated-patch voltage-clamp recordings were used. Whole-cell recording techniques were used for measuring spontaneous inhibitory postsynaptic currents (sIPSCs) and optically-evoked IPSCs (oIPSCs) while perforated-patch recordings were used for the calcium current experiment. Voltage-clamp recordings were performed using a Multiclamp 700B amplifier or Axopatch 200A (Molecular Devices, San Jose, CA), Digidata 1440A interface and pClamp 10 software (Molecular Devices, San Jose, CA). Membrane capacitance (pF) was obtained by digital cancelation of whole-cell capacitance transients. Recordings for sIPSCs and oIPSCs were made from a holding potential of –60 mV, low-pass filtered at 2 kHz and digitized at 10 kHz. Patch pipettes were pulled from glass capillary tubing (KG-33, 1.5 mm OD, King Precision Glass, Claremont, CA) on a Flaming/Brown P-87 pipette puller (Sutter Instrument^®^, Novato, CA) with resistance 4–8 MΩ when filled with a pipette solution. The internal pipette solution for whole-cell recordings contained (mM): CsCl, 130; ethylene glycol-bis (β-aminoethyl ether) N,N,N′,N′-tetraacetic acid (EGTA), 10; MgCl_2_, 2; HEPES, 10; Mg-ATP, 4; GTP, 0.1; pH 7.2 with CsOH; 295–300 mOsm, as described previously (Murchison and Griffith, 1996). For perforated-patch recordings, the internal solution contained (mM): CsAc, 120; CsCl, 15; HEPES, 20; EGTA, 0.4; and amphotericin B (240 mg/ml), pH 7.2, 290–300 mOsm. AMI (Sigma, St. Louis, MO) was prepared as a 10 mM stock solution in distilled water and diluted in external bath solution for each experiment just before use.

#### Measuring Calcium Currents From Rat BF Neurons

Acutely dissociated neurons from juvenile rats were used for these studies, as describe above in section “Acutely Dissociated Neurons and Reduced Synaptic Preparation.” For experiments studying calcium currents, barium (2 mM) was used as the charge carrier and voltage-clamp was obtained by the perforated-patch method using amphotericin B (240 mg/ml) (Murchison et al., 2009). Amphotericin B was included in the patch pipette and neurons were allowed to dialyze (∼4 min after maximum perforation) prior to recording. Cells were held at –70 mV and a test pulse (to –15 mV, 90 ms duration) was applied every 20 s. Barium replaces calcium as the charge carrier in the bath recording solution to prevent calcium-dependent processes and was used throughout the experiment as described in our previous studies (Murchison et al., 2009). Increasing concentrations of AMI were applied by focal drug barrel until a stable current was obtained. Currents were measured from 0 mV to peak, and the percent inhibition of HVA calcium current by AMI was plotted.

#### Optically-Induced Current Recordings and Quantal Content Analysis

Neurons in the reduced synaptic preparation were mechanically dissociated from small sections of basal forebrain as describe above and dispersed onto a recording chamber. Optical stimulation was applied using 5-ms duration of 470 nm light at 15 s intervals using an optogenetic illumination system (DC2100, Thor Labs, Ann Arbor, MI) and 50–100 traces of optically-induced inhibitory postsynaptic currents (oIPSCs) were recorded. For the quantal content analysis, minimal stimulation was used in order to ensure a sufficient number of failures. LED intensity was controlled by adjusting the drive current (1 mA-1A). Power density was calculated by dividing the measured power by the measured illuminated area using a PM200 optical power and energy meter (ThorLabs, Ann Arbor, MI) connected to an S120C photodiode sensor (ThorLabs, Ann Arbor, MI). Initial measurements using a 400 μm cannula revealed a maximum power density of approximately 10 mW/mm^2^, and a power density of 1.76 mW/mm^2^ corresponding to 100 mA drive current. For the quantal content analysis, using the method of failures (m = ln(N/N_0_)) in the Poisson model, quantal content (m) was calculated where N is total number of stimuli, and N_0_ is the number of failures where stimulation failed to evoke a synaptic response.

#### Electrophysiological Analysis

Mini Analysis (6.0.7; Synaptosoft, Decatur, GA) and Clampfit 10 (Molecular Devices, San Jose, CA) software were employed for the analysis. The evoked response was calculated as the peak current over 20 ms from the time when response begins. Mean data were compared using a paired *t*-test with significance determined by *p* < 0.05 (GraphPad, San Diego, CA). Values were expressed as mean ± S.E.M.

### Calcium Imaging

Standard fura-2 ratiometric microfluorimetry (Murchison and Griffith, 1998, 2007; Murchison et al., 2009) was used to measure intracellular calcium changes in enzyme-treated mouse BF neurons. Neurons were acutely dissociated and dispersed onto a cover slip and were loaded with ratiometric fluorescent Ca^2+^-indicator fura-2AM (1 μM, Molecular Probes, Eugene, OR) from the bath for 12 min in the presence of 10 μM pluronic acid (Invitrogen, Carlsbad CA) followed by 40–45 min washout to allow for fura de-esterification. The resulting fluorescence is equivalent to ∼100 μM impermeable salt of fura-2 (pentapotassium salt, fura-2 K^+^_5_). Fluorescence was recorded with 340 nm and 380 nm excitation and the 340/380 ratio, which increases with increasing [Ca^2+^] was calculated. In order to achieve an adequate sampling rate for the fura-2 data shown in Figure 2, the pixels were binned and the fluorescent intensities were measured from a region of interest, centered on and smaller than the cell soma. Intracellular calcium concentrations ([Ca^2+^]_i_) were estimated as described previously (Murchison and Griffith, 1998). Ca^2+^-transients (Δ[Ca^2+^]_i_) were triggered by focal picospritzer (Picospritzer II, General Valve, Fairfield NJ) application of recording solution with elevated [K^+^] (20 mM) solution (High [K^+^]). Fluorescent signals were recorded using a Sutter lambda DG-4 excitation switcher (Sutter Instrument^®^, Novato, CA), a Hamamatsu ORCA flash2.8 CCD camera (Hamamatsu Photonics, Bridgewater Township, NJ), an Olympus IX70 fluorescent scope and Metafluor imaging software (Molecular Devices, San Jose, CA).

### Statistical Analysis

Comparisons were performed using a paired *t*-test with significance determined by *p* < 0.05 (GraphPad, San Diego, CA). Values are expressed as means ± S.E.M. For wheel-running statistical analyses, raw data were analyzed using a two-way ANOVA to determine the significance of age and genotype effects on circadian properties and quantitative parameters of the activity rhythm. In each case, differences in circadian behavior were considered significant at *p* < 0.05.

## Results

### Optogenetic Aging Model and Reduced Synaptic Preparation

**FIGURE 1:**
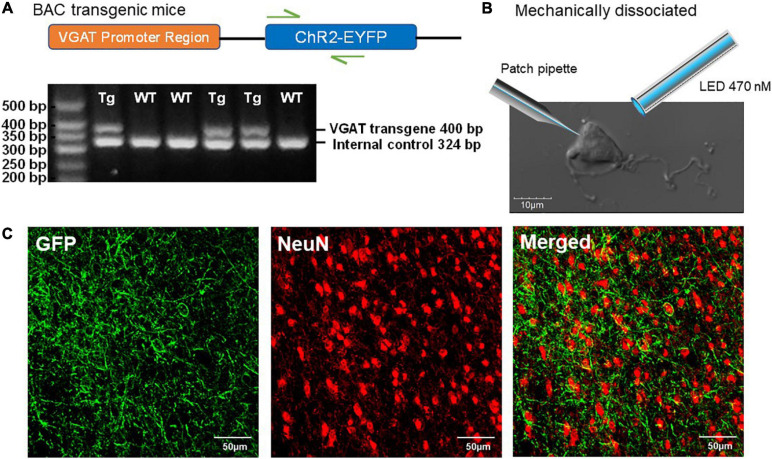
Characterization of ChR2-EYFP expression in VGAT-ChR2(H134R)-EYFP transgenic mice. **(A)** Schematic representation of VGAT-ChR2-EYFP transgene (top) and an example of PCR gel electrophoresis (bottom). The first lane is the DNA size standard and the fragment size is given in base pairs on the left. The genotypes are described at the top of each lane. **(B)** The representative mechanically dissociated BF neuron with the illustrations of patch pipette and 470-nm LED light stimulation. **(C)** Confocal images of mouse BF slice show strong ChR2-EYFP expression in GABAergic neurons of VGAT-ChR2(H134R)-EYFP transgenic mice.

We recently demonstrated that the expression of ChR2 in mice carrying the VGAT-ChR2-EYFP transgene is functionally maintained across aging, thus this mouse line is useful for aging neuroscience (Montgomery et al., in submission). In mice hemizygous for VGAT-ChR2-EYFP BAC transgene, the ChR2-EYFP fusion protein is expressed in GABAergic neuronal populations by the mouse vesicular GABA transporter (VGAT) promoter on the BAC transgene (Figure 1A), which enables specific optical stimulation of GABAergic neurons. Whole brain coronal images showed strong ChR2-EYFP expression in a section of the BF (Figure 1C).

Our “reduced synaptic preparation” was designed to investigate synaptic physiology during aging with greater efficiency than in an aged slice preparation. This reduced preparation consists of acutely dissociated neurons that are not exposed to the usual proteolytic enzyme treatment and therefore retain functional presynaptic inputs on somas and proximal dendrites (Akaike and Moorhouse, 2003). Figure 1B shows an enzyme-free, acutely dissociated BF neuron and an illustration of the patch pipette and optogenetic light-stimulation.

### Effects of AMI on Calcium

**FIGURE 2:**
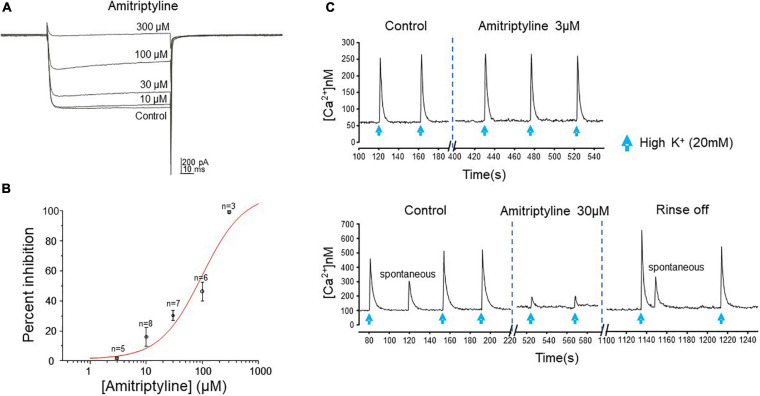
AMI effect on calcium current and intracellular calcium transients. **(A)** Whole-cell perforated-patch voltage-clamp recordings of calcium currents from a rat BF neuron show reduced current amplitude in the presence of increasing concentrations of AMI. Currents were elicited by a depolarizing test pulse to –15 mV (V_h_ = –70 mV, 20 s between traces). **(B)** Cumulative concentration-response curve for AMI inhibition of calcium current in rat BF neurons. Percent inhibition of calcium current observed at five different concentrations (3, 10, 30, 100, and 300 μM) was plotted (± S.E.M.), yielding an EC_50_ of 98.02 μM. **(C)** Fura-2 microfluorimetric recordings of intracellular calcium transients evoked by picospritzer application of 20 mM K^+^ (blue arrows, top panel: 600 ms, lower panel: 400 ms duration) show the effects of AMI on mouse BF neurons. The upper figure shows no effect of 3 μM AMI on the amplitude of calcium transients. In a different cell, the lower figure shows reversible inhibition of calcium transient amplitude by 30 μM AMI (*n* = 4). Spontaneous Ca^2+^ events are seen in the lower panel, as well.

The effect of AMI on whole-cell high-voltage-activated (HVA) calcium currents was investigated by applying a series of concentrations to acutely dissociated BF neurons from juvenile rats. The effect of AMI on calcium current inhibition was tested at the following five concentrations: 3 μM (*n* = 5), 10 μM (*n* = 8), 30 μM (*n* = 7), 100 μM (*n* = 6) and 300 μM (*n* = 3) with AMI inhibiting calcium currents by 1.87 ± 0.65%, 16.02 ± 6.37%, 30.25 ± 3.42%, 46.29 ± 6.31% and 99.26 ± 0.74%, respectively (Figure 2A). Increasing concentrations were applied on the same neuron and the concentration-response curve for AMI is shown in Figure 2B. It is well known that high concentrations of AMI block voltage-gated Ca^2+^ channels (see Introduction), so the purpose of this experiment was to identify those concentrations of AMI below significant Ca^2+^ inhibition. The curve has a Hill coefficient of 1.2 and an EC_50_ (effective concentration for 50% inhibition) of approximately 100 μM.

To confirm that AMI acts in a similar manner on calcium signaling in the mouse, we used the ratiometric calcium-sensitive fluorescent probe fura-2 and induced robust intracellular calcium transients in acutely dissociated BF neurons by applying 20 mM KCl (High [K^+^]) by focal picospritzer. High [K^+^] depolarizes the membrane and activates voltage-gated Ca^2+^ channels. We then tested the ability of AMI to inhibit these transients (Figure 2C). AMI (30 μM) significantly inhibited high [K^+^]-induced calcium transients by approximately 72% (Figure 2C, bottom panel, control: 238 ± 58.67 nM, and 30 μM AMI: 65.5 ± 19.85 nM, *n* = 4, ^∗^*p* < 0.05). On the other hand, 3 μM AMI had no effect on transient amplitude which is consistent with the previous rat BF data showing very little inhibition of Ca^2+^ currents at that concentration. We next determined the concentration-response relationship for AMI inhibition of synaptic transmission.

### AMI Decreases Inhibitory Synaptic Transmission in Mouse BF Neurons

To study synaptic transmission in mouse BF neurons, we used whole-cell voltage-clamp and the reduced synaptic preparation previously described in rats (Griffith et al., 2014). Briefly, this preparation utilizes acutely dissociated neurons, without enzyme treatment, resulting in isolated cells with functional synapses. The predominant spontaneous synaptic events in this preparation are GABAergic IPSCs. These currents are easily identified, from the very infrequent spontaneous EPSCs, by their larger amplitude (using Cl^–^ filled electrodes, V_h_ = −60 mV) and long-time course compared to the smaller and very fast spontaneous EPSCs (Griffith et al., 2014). We visually identified and measured spontaneous IPSCs in both WT and VGAT mice.

**FIGURE 3:**
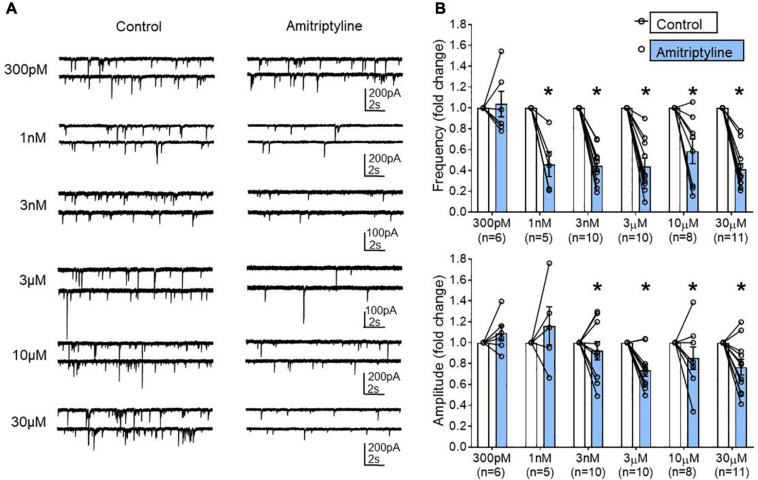
Concentration-dependent changes in the frequency and amplitude of spontaneous IPSCs using a reduced synaptic preparation of BF neurons. **(A)** Representative whole-cell voltage-clamp recordings (Vh = –60 mV) from young BF neurons in control (left) and AMI (right). Concentrations are shown to the left and each neuron only received one concentration of AMI. Frequency and amplitude of sIPSCs were decreased in a concentration-dependent manner by application of AMI. **(B)** Summary data showing the normalized frequency change in AMI (top, 300 pM: 1.038 ± 0.123, 1 nM: 0.460 ± 0.120, 3 nM: 0.44 ± 0.06, 3 μM: 0.44 ± 0.08, 10 μM: 0.58 ± 0.12, 30 μM: 0.41 ± 0.06) and normalized amplitude change in AMI (bottom, 300 pM: 1.09 ± 0.07, 1 nM: 1.11 ± 0.16, 3 nM: 0.92 ± 0.09, 3 μM: 0.74 ± 0.06, 10 μM: 0.85 ± 0.11, 30 μM: 0.77 ± 0.07). Results are presented as mean ± SEM. Individual cells (n) were paired for control and AMI (paired *t*-test, *p < 0.05). *N* = 23 mice.

Six different concentrations of AMI were tested in young mouse BF neurons: 300 pM (*n* = 6), 1 nM (*n* = 5), 3 nM (*n* = 10), 3 μM (*n* = 10), 10 μM (*n* = 8), and 30 μM (*n* = 11). Figure 3A shows whole-cell voltage-clamp recordings of sIPSCs from the BF neurons in paired control and AMI for each concentration (V_h_ = –60 mV). The frequency of sIPSCs was significantly reduced in the presence of AMI at almost all concentrations tested (Figure 3B, top). The amplitude of sIPSCs was also significantly reduced by most concentrations of AMI (Figure 3B, bottom, paired *t*-test, ^∗^*p* < 0.05). We performed the remainder of the experiments using 3 μM AMI because this concentration had minimal effect on calcium currents and transients but significantly reduced the frequency of sIPSCs. Furthermore, 3–6 μM AMI is considered a clinically relevant concentration for depression treatment (Glotzbach and Preskorn, 1982; Lavoie et al., 1990; Baldessarini, 2006).

### AMI Decreases Spontaneous IPSCs in Both Young and Aged BF Neurons

The remaining experiments used both young (7–10 mo.) and aged (23–25 mo.) VGAT-ChR2-EYFP mice. In addition, these cohorts were monitored for wheel-running behavior in order to confirm the well-known age-related changes in patterns of circadian entrainment (Figure 7).

**FIGURE 4:**
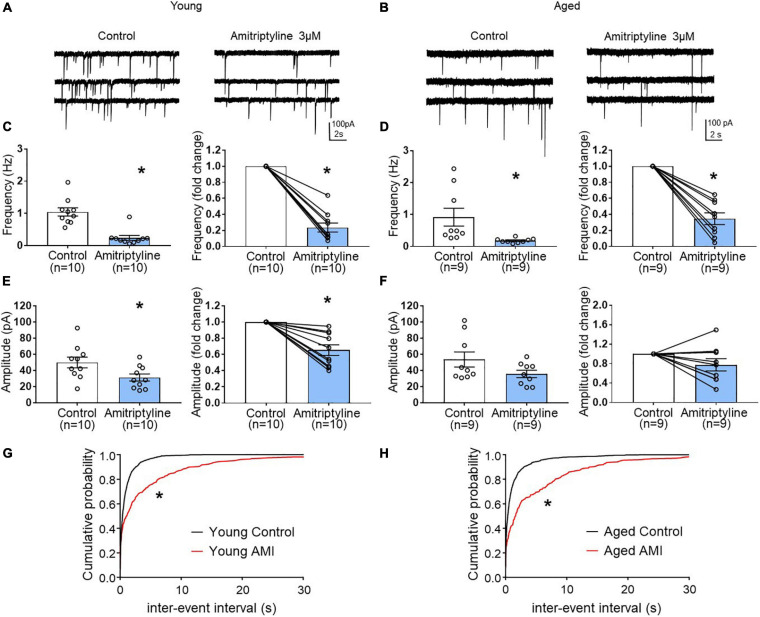
The effect of AMI (3 μM) on spontaneous IPSC frequency and amplitude in BF neurons from young and aged VGAT mice using the reduced synaptic preparation. Representative whole-cell voltage-clamp recordings (V_h_ = –60 mV) from a young **(A)** and aged **(B)** BF neuron in control (left) and 3 μM AMI (right). **(C)** Scatter plots with mean ± SEM of frequency (left panel, 1.05 ± 0.13 Hz for control, 0.24 ± 0.07 Hz in 3 μM AMI, paired *t*-test, *p < 0.05) and graph of normalized fold change in frequency (right panel, 0.24 ± 0.06 for 3 μM AMI, paired *t*-test, *p < 0.05) show that AMI decreases the frequency of sIPSCs in young BF neurons. **(D)** Scatter plots with mean ± SEM of frequency (left, control: 0.92 ± 0.28 Hz, 3 μM AMI: 0.18 ± 0.02 Hz, paired *t*-test, *p < 0.05) and graph of normalized fold change in frequency (right, 3 μM AMI: 0.35 ± 0.07, paired *t*-test, *p < 0.05) show that AMI decreases the frequency of sIPSCs in aged BF neurons. **(E)** Scatter plots with mean ± SEM of amplitude (left panel, 50.06 ± 6.62 pA in control, 31.37 ± 4.44 pA in 3 μM AMI, paired *t*-test, *p < 0.05) and graph of normalized fold change in amplitude (right panel, 0.65 ± 0.07 in 3 μM AMI, paired *t*-test, *p < 0.05) show that AMI decreases the amplitude of sIPSCs in young BF neurons. **(F)** Scatter plots with mean ± SEM of amplitude (left, control: 53.75 ± 9.32 pA, 3 μM AMI: 35.9 ± 4.58 pA, paired *t*-test, *p* = 0.07) and graph of normalized fold change in amplitude (right, 3 μM AMI: 0.78 ± 0.13, paired *t*-test, *p* = 0.11) show that AMI did not significantly alter the amplitude of sIPSCs in aged BF neurons. **(G)** Cumulative probability plots of sIPSC inter-event intervals in young mouse BF neurons (*n* = 10). **(H)** Cumulative probability plots of sIPSC inter-event intervals in aged mouse BF neurons (*n* = 9). In AMI, the cumulative probability curves were significantly different from control (*p < 0.01, K-S test). *N* = 5 young and *N* = 5 aged mice.

In young neurons, both mean frequency and mean amplitude of sIPSCs were significantly reduced by 3 μM AMI. Figure 4A shows whole-cell voltage-clamp recordings of sIPSCs from young BF neurons in control and AMI (V_h_ = −60 mV). The mean sIPSC frequencies were 1.05 ± 0.13 Hz for control and 0.24 ± 0.07 Hz in AMI (left in Figure 4C, paired *t*-test, ^∗^*p* < 0.05), and the inhibition normalized to control frequency is shown on the right (paired *t*-test, ^∗^*p* < 0.05). For sIPSC amplitude, the means were 50.06 ± 6.62 pA in control and 31.37 ± 4.44 pA in AMI (left in Figure 4E, paired *t*-test, ^∗^*p* < 0.05), and the normalized change is shown on the right (paired *t*-test, ^∗^*p* < 0.05). Figures 4G,H show the cumulative probability curves of young and aged sIPSC frequencies in control (black) and in 3 μM AMI (red). These curves are significantly different by the Kolmogorov-Smirnov test (^∗^*p* < 0.01).

The same experiments were repeated in aged BF neurons (Figures 4B,D,F,H). Whole-cell voltage-clamp recordings (V_h_ = −60 mV) from the same neuron are shown in Figure 4B. Similar to young mice, the mean frequency of sIPSCs in aged BF neurons was significantly reduced by AMI (Figure 4D left, control: 0.92 ± 0.28 Hz, 3 μM AMI: 0.18 ± 0.02 Hz, paired *t*-test, ^∗^*p* < 0.05). At the right on Figure 4D, the normalized difference between control and AMI also was significant (paired *t*-test, ^∗^*p* < 0.05). Although the mean amplitude of sIPSCs in the aged group was reduced in the presence of 3 μM AMI, the change was not significant (left in Figure 4F, control: 53.75 ± 9.32 pA, AMI: 35.9 ± 4.58 pA, paired *t*-test, *p* = 0.07). The normalized change in the sIPSC amplitude between control and AMI in aged BF neurons also was not significant (Figure 4F right, paired *t*-test, *p* = 0.11). Despite the lack of significant effect of 3 μM AMI on aged sIPSC amplitude, the reduction of both young and aged sIPSC frequency suggests that AMI remains efficacious during aging.

### AMI Decreases Light-Induced oIPSCs in Both Young and Aged BF Neurons

**FIGURE 5:**
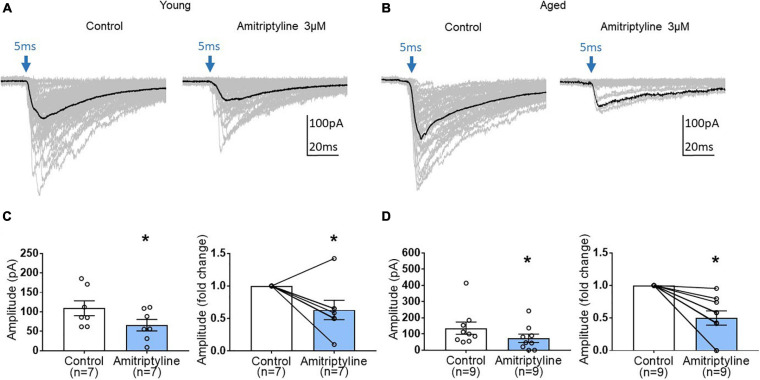
The effect of AMI (3 μM) on light-induced optical IPSC (oIPSC) amplitude in BF neurons of young and aged VGAT mice in the reduced synaptic preparation. **(A,B)** Superimposed (50 traces) whole-cell voltage-clamp recordings (V_h_ = –60 mV) of evoked oIPSCs from a young **(A)** and an aged **(B)** BF neuron in control (left) and 3 μM AMI (right). The evoking stimulus was a 5 ms pulse of 470 nm light at constant intensity (blue arrow). The black trace represents the mean oIPSC and failures were not included in the calculation. **(C)** Scatter plots with mean data ± SEM of oIPSC amplitude (left panel, control: 109.3 ± 19.05 pA, 3 μM AMI: 65.85 ± 14.75 pA, paired *t*-test, *p < 0.05) and normalized fold change of oIPSC amplitude (3 μM AMI: 0.63 ± 0.15, paired *t*-test, *p < 0.05) show that AMI significantly decreases the amplitude of light-induced oIPSCs in young BF neurons compared to control. **(D)** Scatter plots with mean ± SEM of oIPSC amplitude (left panel, control: 136 ± 37.88 pA, 3 μM AMI: 73.8 ± 25.83 pA, paired *t*-test, *p < 0.05) and normalized fold change of oIPSC amplitude (3 μM AMI: 0.50 ± 0.11, paired *t*-test, *p < 0.05) show that AMI significantly decreases the amplitude of light-induced oIPSCs in aged BF neurons. *N* = 5 young and *N* = 4 aged mice.

To examine further synaptic mechanisms of AMI on inhibitory synaptic transmission, light-induced oIPSCs were measured before and after treatment with 3 μM AMI in BF neurons from young and aged VGAT mice. As seen in superimposed voltage-clamp (Vh = −60 mV) recordings in Figure 5A, AMI significantly decreased the amplitude of light-induced (470 nm, 5 ms, constant intensity at blue arrow) oIPSCs in young BF neurons (left in Figure 5C, control: 109.3 ± 19.05 pA, AMI: 65.85 ± 14.75 pA, paired *t*-test, ^∗^*p* < 0.05) and the normalized amplitude (Figure 5C right, paired *t*-test, ^∗^*p* < 0.05). Parallel experiments on oIPSCs using BF neurons from aged VGAT mice are seen in Figures 5B,D. Similarly, the amplitude of light-induced oIPSC (Figure 5B) was significantly decreased by 3 μM AMI (left in Figure 5D, control: 136 ± 37.88 pA, 3 μM AMI: 73.8 ± 25.83 pA, paired *t*-test, ^∗^*p* < 0.05). The normalized amplitude change is shown on the right of Figure 5D (paired *t*-test, ^∗^*p* < 0.05). In Figures 5A,B, the superimposed gray traces represent 50 repetitions of the stimulus and the black trace is the average. Failures were not included in the measurement of the mean amplitudes.

**FIGURE 6:**
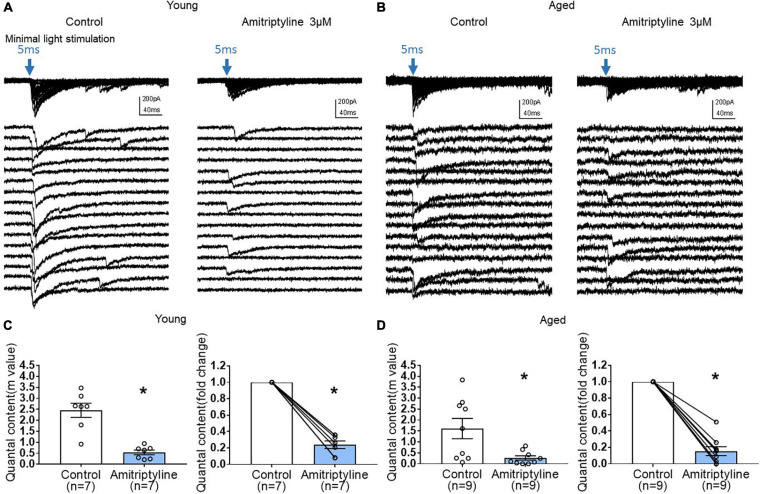
AMI (3 μM) decreases quantal content of inhibitory synaptic transmission in both young and aged BF neurons in the reduced synaptic preparation of VGAT mice. **(A,B)** Representative whole-cell voltage-clamp recordings (V_h_ = –60 mV) of oIPSCs superimposed (top) and expanded (below) in young and aged mice (left: control, right: 3 μM AMI). Blue arrow indicates 470nm light stimulation with 5-ms duration and constant minimal intensity. The expanded traces show 16 representative sweeps of the 50–100 stimulus repetitions recorded for each cell. **(C)** Scatter plots showing mean quantal content (m) in control and 3 μM AMI at left (*m* = 2.46 ± 0.32 for control, 0.54 ± 0.11 for 3 μM AMI, paired *t*-test, *p < 0.05) and on the right panel showing normalized fold change of quantal content by AMI in young BF neurons (3 μM AMI: 0.24 ± 0.05, paired *t*-test, *p < 0.05). **(D)** Graphs showing quantal content (m) in control and 3 μM AMI in aged BF neurons (left, *m* = 1.61 ± 0.47 for control, 0.26 ± 0.10 for 3 μM AMI, paired *t*-test, *p < 0.05) and normalized fold change of quantal content by 3 μM AMI in aged BF neurons (right, 3 μM AMI: 0.15 ± 0.05, paired *t*-test, *p < 0.05). Quantal content (m) of inhibitory synaptic transmission was significantly decreased by application of AMI both in young and aged BF neurons. Bars represent mean ± SEM. *N* = 5 young and *N* = 4 aged mice.

To investigate further a possible presynaptic contribution of AMI to reduced oIPSCs, we performed quantal analysis using the method of failures. Quantal content (m) was determined by m = ln(N/N_0_) as described in the methods. The power of this analysis is that no assumptions concerning synaptic number are necessary. Figures 6A,B show that minimal light stimulation (5 ms, blue arrow) generates both light-evoked currents and failures in both young and aged neurons. In the presence of AMI (3 μM), the number of failures increased in both age groups. Analysis revealed that quantal content was significantly decreased by 3 μM AMI in both young (left in Figure 6C, m = 2.46 ± 0.32 for control and 0.54 ± 0.11 for AMI) and aged (left in Figure 6D, m = 1.61 ± 0.47 for control and 0.26 ± 0.10 for AMI; paired *t*-test, ^∗^*p* < 0.05). The normalized fold change in quantal content between control and AMI is shown to the right in Figures 6C,D (paired *t*-test, ^∗^*p* < 0.05).

### Effect of Age on Daily Circadian Wheel-Running Entrainment

**FIGURE 7:**
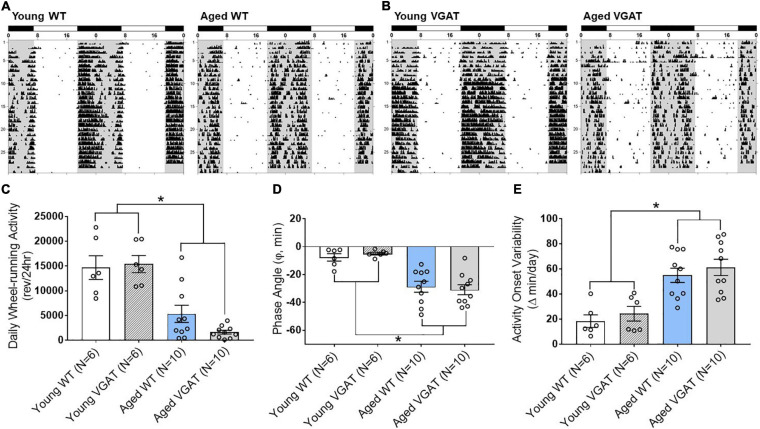
Effects of aging on light-dark entrainment and other properties of the circadian rhythm of wheel-running activity in WT and VGAT mice. Representative records of circadian wheel-running behavior in WT **(A)** and VGAT **(B)** mice comparing the activity rhythms of young (left) and aged (right) animals during entrainment to LD 12:12. Actograms are double-plotted over a 48-h period. Each dot on the actogram represents 50-wheel rotations per 10-min window. The closed bars at the top and shading on the records signify the timing of the dark phase in the LD 12:12 cycle. **(C)** Total daily wheel-running activity of young and aged mice for each genotype (WT, VGAT). Bars depict average wheel revolutions per 24 h (± SEM) [Young WT: 14,695 ± 2,387, Young VGAT: 15,410 ± 1,740, Aged WT: 5,358 ± 1,744, Aged VGAT: 1,655 ± 382.7, two-way ANOVA, age effect; *F*(1, 28) = 51.02, *p < 0.0001, genotype effect; *F*(1, 28) = 0.85, *p* = 0.36]. **(D)** Phase angle between daily activity onsets and lights-off during LD 12:12 entrainment in young and aged WT and VGAT mice. Bars represent the mean (± SEM) phase angle of entrainment (Ψ) to LD 12:12 in minutes. Negative values indicate that daily onsets of activity occur after lights-off whereas positive values denote that activity onsets precede the end of the light phase [Young WT: –7.70 ± 2.63 min, Young VGAT: –4.97 ± 0.97 min, Aged WT: –28.76 ± 3.94 min, Aged VGAT: –30.93 ± 3.57 min, two-way ANOVA, age effect; *F*(1, 28) = 41.74, *p < 0.0002, effect of genotype; *F*(1, 28) = 0.01, *p* = 0.95]. **(E)** Absolute day-to-day variability in daily onsets of activity. Absolute differences in the timing of activity onsets on successive days were analyzed in individual animals and bars represent mean (± SEM) determinations in minutes [Young WT: 18.38 ± 5.03 min, Young VGAT: 24.33 ± 5.84 min, Aged WT: 54.88 ± 5.70 min, Aged VGAT: 61.21 ± 6.49 min, two-way ANOVA, age effect; *F*(1, 28) = 33.29, *p < 0.0001, effect of genotype; *F*(1, 28) = 0.93, *p* = 0.34]. There were no significant differences between genotypes. *N* = 6 young and *N* = 10 aged mice for each genotype.

For our electrophysiology experiments described above using VGAT mice (Figures 4–6), we utilized the well characterized circadian wheel-running entrainment model to validate our optogenetic aging model. During exposure to LD 12:12, entrainment of the activity rhythm was observed in all WT and VGAT mice. Representative actograms of young and aged animals from both WT (Figure 7A) and VGAT (Figure 7B) mice are shown and constitute the raw data from which the analysis (Figures 7C–E) was conducted. During entrainment to LD 12:12, young and aged mice of both genotypes were distinguished by robust differences in the total amount of daily wheel-running activity. Daily activity levels (wheel revolutions/24 h) were significantly and up to 8-fold greater in young WT and VGAT mice compared to aged subjects (Figure 7C, two-way ANOVA, age effect; F (1,28) = 51.02, ^∗^*p* < 0.0001). No effect of genotype was observed in daily wheel-running activity [two-way ANOVA, genotype effect; *F*(1, 28) = 0.85, *p* = 0.36]. In both WT and VGAT mice, the young and aged cohorts were characterized by clear differences in their patterns of circadian entrainment. In the young mice, daily onsets of activity occurred near or shortly after lights-off such that the average phase angle (Ψ) between the activity onsets and the offset of the photoperiod was −7.70 ± 2.63 for WT and −4.97 ± 0.97 min for VGAT (Figure 7D). In contrast, the activity rhythms of aged mice were distinguished by an altered phase angle of entrainment to LD 12:12 such that their daily onsets of activity were delayed and occurred at later times relative to young animals, commencing more than 20 min after lights-off for most animals (Figures 7A,B,D). In the aged WT and VGAT groups, the average phase angles were –28.76 ± 3.94 and –30.93 ± 3.57 min, respectively. These values were significantly greater than those observed in young animals for each genotype [Figure 7D, two-way ANOVA, age effect; *F*(1, 28) = 41.74, ^∗^*p* < 0.0001]. There was no effect of genotype on phase angle of activity [two-way ANOVA, effect of genotype; *F*(1, 28) = 0.01, *p* = 0.95]. In addition to the delayed onsets of daily activity, aged WT and VGAT animals showed unstable patterns of entrainment to LD 12:12 as indicated by high variability in the timing of their activity onsets between successive days (Figures 7A,B,E). During exposure to LD 12:12, the activity onsets in individual aged WT and VGAT mice occurred at (earlier or later) times that differed on average, respectively, by 54.88 ± 5.70 and 61.21 ± 6.49 min from the preceding day. Whereas the average day-to-day variability in activity onset times of young animals was only 18.38 ± 5.03 (WT) and 24.33 ± 5.84 (VGAT) minutes (Figure 7E). The values for day-to-day variation in the onsets of activity in the aged WT and VGAT groups were significantly greater [two-way ANOVA, age effect; *F*(1, 28) = 33.29, ^∗^*p* < 0.0001] than those observed in their young counterparts. No significant difference in day-to-day variability in activity onset times was observed between age-matched WT and VGAT mice [two-way ANOVA, effect of genotype; *F*(1, 28) = 0.93, *p* = 0.34].

## Discussion

In the present study, we show that therapeutic concentrations of AMI decrease spontaneous and light-evoked IPSCs in BF neurons at concentrations below those that block HVA Ca^2+^ currents. Importantly, this synaptic inhibition is preserved during aging. The therapeutic concentrations of AMI for the treatment of depression are 3–6 μM (Glotzbach and Preskorn, 1982; Lavoie et al., 1990), while lower concentrations are also effective for chronic pain and migraine (Riediger et al., 2017; Urquhart et al., 2018; Suga et al., 2019). These therapeutic actions of AMI are maintained in the elderly (Barber and Gibson, 2009). Our data suggest that inhibition of GABAergic synaptic transmission may be one of the mechanisms by which AMI acts therapeutically across aging. In our model, a low concentration of AMI, in the therapeutic range, reduced inhibitory synaptic transmission, whereas higher concentrations were required to block HVA Ca^2+^ currents and high K^+^ induced intracellular Ca^2+^ transients.

AMI has a broad profile of pharmacological actions, including inhibition of biogenic amine reuptake, antagonism of serotonin, muscarinic, α1-adrenergic, and histamine receptors (Richelson, 1979; Kachur et al., 1988; Pandey et al., 2010; Nojimoto et al., 2010; Liu et al., 2012). It also acts to block Ca^2+^ and Na^+^ channels (Brau et al., 2001; Nicholson et al., 2002; Yan et al., 2010; Wu et al., 2012). Additional actions of AMI are described for the NMDA receptor ion channel complex that may contribute to efficacy against neuropathic pain, including the enhancement of Ca^2+^-dependent receptor desensitization and acting as an open channel blocker of NMDARs (Stepanenko et al., 2019). The enhancement of NMDA receptor desensitization shows a strong dependence on extracellular [Ca^2+^] concentrations with an e-fold shift in IC_50_ achieved by a [Ca^2+^] change of 0.63 mM (Stepanenko et al., 2019). These actions on the NMDA receptor provide a likely mechanism for results showing that AMI inhibited the NMDA-dependent LTP in the hippocampus (Watanabe et al., 1993). Also, the Stepanenko study reported an IC_50_ value of 4.9μM in [2 mM] Ca^2+^ that is very similar to the concentration used in our experiments (Figures 4–6) and suggests that AMI may mediate important actions to regulate neurotransmitters by a mechanism that does not involve voltage-gated channels. However, a slight inhibition of presynaptic I_Ca_ cannot be ruled out, as concentrations of AMI as low as 1 μM have been shown to modestly inhibit I_C__a_ in trigeminal ganglion neurons (Wu et al., 2012).

The probability of neurotransmitter release is directly proportional to the Ca^2+^ change within the synaptic terminal (Zucker, 1993; Wu and Saggau, 1997) and AMI regulation of synaptic transmission by this mechanism has been shown using mechanically isolated medullary dorsal horn neurons (Cho et al., 2012). Here, glycinergic neurotransmission is enhanced by higher concentrations of AMI (30 μM) through a suggested presynaptic mechanism to increase neurotransmitter release. These authors provide evidence that AMI increases spontaneous miniature glycinergic IPSCs onto acutely isolated neurons by increasing the interterminal Ca^2+^ concentration, which might be mediated by the Ca^2+^ release from the Ca^2+^ stores rather than the Ca^2+^ influx from the extracellular space (Cho et al., 2012). Chronic AMI (and other antidepressant) treatments have been shown to affect synaptic plasticity in the hippocampus (Zarei et al., 2014; Zhang et al., 2020) with fewer studies focusing on the acute actions of AMI to regulate synaptic transmission (Watanabe et al., 1993). It is interesting to speculate that, in our study, AMI may decrease the frequency of sIPSCs by a mechanism that regulates intraterminal Ca^2+^ stores. We did not measure directly the change in intraterminal Ca^2+^ concentrations induced by AMI, but did not observe a change in somatic baseline [Ca^2+^] on exposure to either 3 or 30 μM AMI (Figure 2). Whereas, presynaptic modulation of neurotransmitter release has been long tied to modulating terminal Ca^2+^ channels, other mechanisms may also come into play to modulate synaptic transmission including fast acting neurotropic factors (Berninger and Poo, 1996; Kovalchuk et al., 2004) and transmembrane regulation of synapses (Tang et al., 2016; Biederer et al., 2017; Han et al., 2021).

Neurotrophins have been implicated in the therapeutic effects of antidepressants with brain derived neurotrophic factor (BDNF) acting as one of the most popular candidates (reviewed Dwivedi, 2009; Colucci-D’Amato et al., 2020). Interestingly, AMI possesses neurotrophic effects by acting directly as an agonist of TrkA and TrkB receptors which BDNF triggers (Jang et al., 2009). AMI may exert neurotrophic effects in primary cortical neurons by activation of a Trk/MAPK signaling pathway and possibly alleviating the loss of synaptic connections under conditions where atrophy and loss of synaptic connections may contribute to progression of neurological diseases (O’Neill et al., 2016). Because BDNF has been proposed as a key transducer of the effects of antidepressants (Björkholm and Monteggia, 2016), it is no surprise that the ability of BDNF to rapidly modulate synaptic transmission has been studied extensively (Kim et al., 1994; Berninger and Poo, 1996; Poo, 2001; Kovalchuk et al., 2004). In the hippocampus, BDNF decreases inhibitory GABAergic transmission (Tanaka et al., 1997; Frerking et al., 1998) whereas BDNF increases excitatory transmission (Tyler et al., 2006). This bidirectional control of synaptic transmission by BDNF would be consistent with the observed effects of AMI if it was acting on synaptic transmission through Trk receptors. While our results showed that acute AMI can decrease synaptic transmission by a presynaptic mechanism to decrease quantal release, in cultured organotypic slices of brainstem noradrenergic neurons in the A1 and A2 area, 1-week treatment of 10 μM AMI increased quantal size and shifted the distribution of events (Chiti and Teschemacher, 2007). It is possible that AMI either acts directly or indirectly through neurotrophins to regulate synaptic transmission depending upon the specific synapses and releasable pools of transmitter (Tyler et al., 2006).

Previously, we found that sIPSC frequency in cholinergic BF neurons is reduced during aging and is associated with cognitive impairment in rats (Griffith et al., 2014). In the present study, AMI decreased the frequency of sIPSCs in young and aged BF neurons of mice. This suggests that AMI could have a negative influence on cognitive function, particularly in the aged. There are numerous studies reporting the effects of AMI on memory and cognitive functions both in humans and in rodent models, however, conclusions differ depending on the experimental conditions. For studies reporting cognitive impairment following AMI treatment, in most cases, a single dose of AMI was administered (Branconnier et al., 1982; Curran et al., 1988; Parra et al., 2002). On the other hand, chronic treatment with AMI demonstrated beneficial effects on cognitive function in clinical and in animal studies (Sternberg and Jarvik, 1976; Staton et al., 1981; Yau et al., 2002; Chadwick et al., 2011). Chronic AMI has been shown to prevent age-related decline and impairment in the water maze task in rats when treatment was initiated at 16 months of age and then tested at 24 months (Yau et al., 2002). These authors suggest that the use of antidepressants may serve as a useful therapeutic approach possibly to ameliorate cognitive impairments in aged individuals.

In our study, we demonstrated that the actions of AMI to reduce synaptic transmission continued during aging in our optogenetic model. We utilized circadian behavioral activity to establish that a parameter of normal aging occurred in this model. Circadian rhythm alterations and disturbances are part of the normal aging process in humans, and these changes in circadian function are further amplified in patients with symptomatic Alzheimer disease (AD) (Witting et al., 1990; Harper et al., 2005). Age- and AD-related changes in circadian rhythms are most pronounced in the sleep-wake cycle (Olivier-Martin et al., 1975; Peter-Derex et al., 2015). Circadian disturbances of sleep-wake rhythms in aging and AD include rhythm fragmentation over the entire cycle with increased nighttime and decreased daytime bouts of activity in conjunction with a decrease in rhythm amplitude (Weitzman et al., 1982; Satlin et al., 1995; Harper et al., 2005; Peter-Derex et al., 2015; Musiek et al., 2018). In AD, these changes in the sleep-wake cycle are often accompanied by a phase delay in peak daily activity that is associated with an increased risk of moderate to severe dementia (Tranah et al., 2011). The effects of aging on rodent circadian rhythms are comparable to those observed in human aging and AD. Common alterations in the circadian activity of aged rodents include alterations in the amplitude and day-to-day accuracy of the activity rhythm and in its entrainment to light-dark cycles (Rusak and Zucker, 1979; Ingram et al., 1982). Consistent with the age-related changes in sleep-wake rhythms reported in humans and rodents, aged WT and VGAT mice in the present study were distinguished by phase delays and increased day-to-day variability in the activity onsets during entrainment to the daily light-dark cycle. Consequently, these findings establish the utility of the VGAT-ChR2 optogenetic mouse as a model to study the relationship between aging and circadian rhythm disturbances. As such, future studies will determine whether this circadian dysfunction precedes cognitive impairment during aging and thus provide a biomarker of preclinical dementia.

Our lab recently developed a new optogenetic aging model which demonstrated that expression of the channelrhodopsin in the VGAT mouse line remained functional throughout the lifespan of the mice and there is no effect of genotype (wild type versus VGAT-ChR2-EYFP) on aging-related physiological and behavioral properties (Montgomery et al., in submission). Here we present data showing that therapeutically relevant concentrations of AMI inhibited sIPSC frequency in the mouse BF neurons regardless of animal age. Given that changes in the frequency reflect changes in probability of neurotransmitter release from presynaptic sites, reduction in sIPSC frequency by AMI suggests that AMI is likely to act presynaptically to decrease the probability of spontaneous GABA release on BF neurons. AMI also significantly decreased the amplitude of sIPSCs in young BF neurons but not quite significantly in aged BF neurons. This indicates that AMI may work on both pre- and post-synaptic sites, perhaps with a greater impact at pre-synaptic sites. This conclusion was supported by the findings that AMI significantly decreased the amplitude of light-induced oIPSCs as well as the quantal content in both young and aged BF neurons without age-related differences. Future studies will be needed to investigate further possible pre-and postsynaptic mechanisms of AMI.

## Data Availability Statement

The original contributions presented in the study are included in the article/supplementary material, further inquiries can be directed to the corresponding author/s.

## Ethics Statement

The animal study was reviewed and approved by Texas A&M University accredited AAALAC committee.

## Author Contributions

EB: conceptualization, design of study, optogenetic data acquisition, calcium imaging, immunohistochemistry, formal analysis, data curation, visualization, and writing-original draft, review and editing. AT: calcium current data acquisition, methodology, and analysis. KM: formal analysis and writing-review and editing. AF: data and animal curation. DE: methodology, circadian behavior data acquisition, writing-original draft, and review and editing. DM: conceptualization, methodology, validation, and writing-review and editing. WG: conceptualization, design of the study, project administration, methodology, validation, writing-original draft, review and editing, supervision, and resources and funding acquisition. All authors contributed to the article and approved the submitted version.

## Conflict of Interest

The authors declare that the research was conducted in the absence of any commercial or financial relationships that could be construed as a potential conflict of interest.
